# Supplementary material for: Mobile applications available in Saudi Arabia for the management of Primary Dysmenorrhea: A quality review and content analysis
Source: PLoS One. 2025 Jun 12;20(6):e0325652. doi: 10.1371/journal.pone.0325652 (PMC12161552; doi:10.1371/journal.pone.0325652)
Supplement: S1 Text — (DOCX) [file pone.0325652.s001.docx]

| **Table: App classification section from MARS tool.** | | | | | | | | | | | | | | | | | | |
| --- | --- | --- | --- | --- | --- | --- | --- | --- | --- | --- | --- | --- | --- | --- | --- | --- | --- | --- |
| App ID | | 01 | 02 | 03 | 04 | 05 | 06 | 07 | 08 | 09 | 10 | 11 | 12 | 13 | 14 | 15 | 16 | n |
| App targets | |  |  |  |  |  |  |  |  |  |  |  |  |  |  |  |  |  |
|  | Increase Happiness/Well-being | X | X | X | X |  | X | X | X | X | X | X | X | X | X | X | X | 15 |
|  | Mindfulness/Meditation/Relaxation | X | X | X | X |  | X | X | X | X | X | X | X | X | X | X | X | 15 |
|  | Reduce negative emotions | X |  | X |  |  | X | X | X |  |  | X | X |  | X | X | X | 10 |
|  | Depression |  |  | X |  |  |  |  | X |  | X |  |  |  | X |  |  |  |
|  | Anxiety/Stress | X |  | X |  |  | X | X | X |  | X | X | X |  | X | X | X | 11 |
|  | Anger |  |  |  |  |  |  |  |  |  |  |  |  |  |  |  |  | 0 |
|  | Behavior Change |  |  |  |  |  |  |  |  |  |  |  |  |  |  |  |  | 0 |
|  | Alcohol /Substance Use |  |  |  |  |  |  |  |  |  |  |  |  |  |  |  |  | 0 |
|  | Goal Setting | X |  | X |  |  |  |  |  |  |  |  | X |  |  |  | X | 4 |
|  | Entertainment | X |  |  |  |  |  |  |  |  |  |  |  | X |  |  |  | 2 |
|  | Relationships | X |  | X |  |  |  |  |  |  |  |  | X |  |  | X |  | 4 |
|  | Physical health | X | X | X | X | X | X | X | X | X | X | X | X | X | X | X | X | 16 |
|  | Other |  |  |  |  |  |  |  |  |  | X |  |  |  |  |  |  |  |
| Theoretical/strategies background | |  |  |  |  |  |  |  |  |  |  |  |  |  |  |  |  |  |
|  | Assessment |  |  |  |  |  |  |  |  | X |  | X | X |  |  | X |  | 4 |
|  | Feedback | X |  |  |  | X |  |  |  |  |  |  | X |  | X | X |  | 5 |
|  | Information/Education | X |  | X | X |  | X | X | X | X | X | X | X | X | X | X | X | 14 |
|  | Monitoring/Tracking | X | X | X | X | X | X | X | X | X | X | X | X | X | X | X | X | 16 |
|  | Goal sitting | X |  | X |  |  |  |  |  |  |  |  | X | X |  |  | X |  |
|  | Advice /Tips /strategies / skills training |  | X | X | X | X | X | X | X | X | X | X | X | X | X |  |  | 13 |
|  | CBT – Behavioral (positive events) |  |  |  |  |  |  |  |  |  |  | X |  |  |  |  |  | 1 |
|  | CBT – cognitive (thought challenging) |  |  |  |  |  |  |  |  |  |  |  |  |  |  |  |  |  |
|  | ACT – Acceptance commitment therapy |  |  |  |  |  |  |  |  |  |  |  |  |  |  |  |  |  |
|  | Mindfulness/ Meditation |  |  | X |  |  | X |  |  |  |  |  | X | X |  | X | X | 6 |
|  | Relaxation | X |  |  |  |  | X | X |  | X |  | X | X |  |  |  | X |  |
|  | Gratitude |  |  |  |  |  |  |  |  |  |  |  |  |  |  |  |  | 0 |
|  | Strengths based |  |  |  |  |  |  |  |  |  |  |  |  |  |  |  |  | 0 |
|  | Other |  |  |  |  |  |  |  |  |  |  |  |  |  |  |  |  | 0 |
| Affiliation | |  |  |  |  |  |  |  |  |  |  |  |  |  |  |  |  |  |
|  | Unknown |  | X |  | X |  |  | X | X | X |  |  |  |  |  |  |  | 5 |
|  | Commercial | X |  | X |  | X |  | X | X | X | X | X | X | X | X | X | X | 13 |
|  | NGO | X | X | X | X | X | X | X | X | X | X | X | X | X | X | X | X | 16 |
|  | Government |  |  |  |  |  |  |  |  |  |  |  |  |  |  |  |  | 0 |
|  | University |  |  |  |  |  |  |  |  |  |  |  |  |  |  |  |  | 0 |
| **Age** | |  |  |  |  |  |  |  |  |  |  |  |  |  |  |  |  |  |
|  | Children (under 12) |  | X |  |  |  |  |  |  |  |  |  |  |  |  |  | X | 2 |
|  | Adolescents (13-17) | X |  |  | X | X | X | X | X |  | X | X | X | X | X | X |  | 12 |
|  | Young Adults (18-25) | X |  | X | X | X | X | X | X |  | X | X | X | X | X | X |  | 13 |
|  | Adults | X |  | X | X | X | X | X |  | X | X | X | X | X | X | X |  | 13 |
|  | General |  | X |  |  |  |  |  |  | X |  | X | X |  | X | X | X |  |
| Technical aspect of app | |  |  |  |  |  |  |  |  |  |  |  |  |  |  |  |  |  |
|  | Allows sharing (Facebook, Twitter, etc.) | X |  |  | X | X |  |  | X | X |  | X | X | X | X | X |  | 10 |
|  | Has an app community | X |  |  |  | X |  |  |  |  |  | X | X | X |  | X |  | 6 |
|  | Allows password-protection | X |  |  |  | X | X |  | X | X |  | X | X | X | X | X | X | 11 |
|  | Requires login |  |  | X |  |  |  |  |  | X | X |  |  | X | X | X | X | 7 |
|  | Sends reminders | X | X | X | X | X | X | X | X | X |  | X | X | X | X | X | X | 15 |
|  | Needs web access to function |  |  |  |  | X |  |  |  |  |  |  |  |  | X |  |  | 2 |
