# Supplementary material for: Mobile applications available in Saudi Arabia for the management of Primary Dysmenorrhea: A quality review and content analysis
Source: PLoS One. 2025 Jun 12;20(6):e0325652. doi: 10.1371/journal.pone.0325652 (PMC12161552; doi:10.1371/journal.pone.0325652)
Supplement: S2 Text — (DOCX) [file pone.0325652.s002.docx]

| **Table: Personal Information, Terms and Conditions, and Disclaimer** | | | | | | | | | | | | | | | | | | |
| --- | --- | --- | --- | --- | --- | --- | --- | --- | --- | --- | --- | --- | --- | --- | --- | --- | --- | --- |
| *Apps asking personal and providing terms and conditions or disclaimers (detailed).* | | | | | | | | | | | | | | | | | | |
| App ID | | 01 | 02 | 03 | 04 | 05 | 06 | 07 | 08 | 09 | 10 | 11 | 12 | 13 | 14 | 15 | 16 | *n* |
| Requires user account | | X |  |  |  | X |  |  |  | X | X |  |  | X |  | X |  | 6 |
| Personal information | |  |  |  |  |  |  |  |  |  |  |  |  |  |  |  |  |  |
|  | Name |  |  |  |  |  | X |  |  |  |  |  | X | X |  | X |  | 4 |
|  | Current weight |  |  |  |  |  |  |  | X |  |  |  |  |  | X |  | X | 3 |
|  | Date of birth / Age | X |  | X |  | X | X |  |  |  |  | X |  | X | X | X | X | 9 |
|  | Height |  |  |  |  |  |  |  |  |  |  |  | X |  | X |  | X | 3 |
|  | Health issues (e.g., anaemia DM, HBP, Thyroid disease, mental issues) |  |  | X |  |  | X |  | X |  |  |  | X |  |  |  | X | 5 |
| Information regarding period | |  |  |  |  |  |  |  |  |  |  |  |  |  |  |  |  |  |
|  | Average cycle length | X |  | X |  |  |  | X |  | X | X | X |  | X | X | X | X | 10 |
|  | Average period (menstruation) length | X |  | X |  |  | X | X | X | X |  | X |  |  | X | X | X | 10 |
|  | Cycle regularity | X |  | X |  |  | X |  | X |  |  | X | X |  |  | X | X | 8 |
|  | User's goal (track cycle…etc.) |  |  | X |  |  | X | X | X |  |  |  | X | X | X | X | X | 9 |
|  | Menstrual symptoms | X |  | X |  |  | X | X | X |  |  |  | X |  |  |  | X | 7 |
|  | Last period date | X |  | X |  |  | X | X | X | X | X | X | X | X |  | X | X | 12 |
| Terms & Conditions / Disclaimer | |  |  |  |  |  |  |  |  |  |  |  |  |  |  |  |  |  |
|  | Presents Terms & Conditions and/or Disclaimer | X |  | X | X |  | X | X | X | X | X | X | X | X | X | X | X | 14 |
|  | Required Terms & Conditions / Disclaimer agreement |  |  | X |  |  |  | X |  |  | X | X | X | X |  | X | X | 8 |
