# Supplementary material for: Mobile applications available in Saudi Arabia for the management of Primary Dysmenorrhea: A quality review and content analysis
Source: PLoS One. 2025 Jun 12;20(6):e0325652. doi: 10.1371/journal.pone.0325652 (PMC12161552; doi:10.1371/journal.pone.0325652)
Supplement: S3 Text — (DOCX) [file pone.0325652.s003.docx]

| **Table: App exercise content** | | | | | | | | | | | | | | | | | | |
| --- | --- | --- | --- | --- | --- | --- | --- | --- | --- | --- | --- | --- | --- | --- | --- | --- | --- | --- |
| *Exercise content assessment and Screening for Contraindications, FITT principle (Frequency, Intensity, Time, and Type)* | | | | | | | | | | | | | | | | | | |
| App ID | | 01 | 02 | 03 | 04 | 05 | 06 | 07 | 08 | 09 | 10 | 11 | 12 | 13 | 14 | 15 | 16 | n |
| Features used to demonstrate or explain an exercise. | |  |  |  |  |  |  |  |  |  |  |  |  |  |  |  |  |  |
|  | Spoken cues. | X |  |  |  |  |  |  |  | X |  |  | X | X |  |  |  | 4 |
|  | Written cues | X | X | X | X |  | X | X | X | X | X | X | X | X | X | X | X | 15 |
|  | Still images/pictures demonstrating an exercise. | X | X | X | X |  |  |  |  | X |  |  | X |  |  |  | X | 6 |
|  | Video/s demonstrating an exercise | X |  |  |  | X |  |  |  | X |  |  | X |  |  |  | X | 5 |
| Screening for contraindications to exercise | |  |  |  |  |  |  |  |  |  |  |  |  |  |  |  |  |  |
|  | Yes |  |  |  |  |  |  |  |  |  |  |  |  |  |  |  |  | 0 |
|  | No | X | X | X | X | X | X | X | X | X | X | X | X | X | X | X | X | 16 |
| FITT | |  |  |  |  |  |  |  |  |  |  |  |  |  |  |  |  |  |
|  | **F**requency of exercise (i.e., how often) |  |  |  |  |  |  | X | X | X |  |  | X |  |  |  | X | 5 |
|  | **I**ntensity of exercise session (i.e., how hard) |  |  |  |  | X |  |  | X |  |  |  | X |  | X |  | X | 5 |
|  | **T**ime of exercise session (i.e., how long) | X |  |  |  |  | X | X | X | X |  |  | X |  |  |  | X | 7 |
|  | **T**ype of exercise session (e.g., yoga, walking) | X | X | X | X |  | X | X | X | X | X | X | X |  | X | X | X | 14 |
|  | Experience level required (e.g., for beginners) |  |  |  |  |  |  |  |  |  |  |  |  |  |  |  | X | 1 |
|  | Equipment required (e.g., Pillow) | X |  |  |  |  |  |  |  | X |  |  |  |  |  |  |  | 2 |
| Types of exercises | |  |  |  |  |  |  |  |  |  |  |  |  |  |  |  |  |  |
|  | Yoga | X |  | X |  |  | X | X | X | X | X | X |  |  | X | X | X | 11 |
|  | Stretching | X | X |  | X |  | X |  | X | X | X |  |  |  | X |  | X | 9 |
|  | Pilates | X |  | X |  |  |  |  |  |  |  |  |  |  |  |  | X | 3 |
|  | Flexibility |  |  |  |  |  |  |  |  |  |  |  |  |  |  |  | X | 1 |
|  | High Intensity Interval Training (HIIT) |  |  |  |  |  |  |  | X |  |  |  |  |  |  |  |  | 1 |
|  | Dancing |  |  |  |  |  |  |  |  |  |  |  |  |  |  |  |  | 0 |
|  | Meditation | X |  |  |  |  |  | X |  | X |  |  | X |  |  | X | X | 6 |
|  | Breathing exercise |  |  |  |  |  | X | X |  |  |  | X | X |  |  |  | X | 5 |
|  | Pelvic Floor / Kegel exercises | X |  |  |  |  |  |  |  |  |  |  |  |  |  |  | X | 2 |
|  | Walking / Jogging / Running | X |  | X |  |  | X | X | X |  | X | X |  |  | X | X | X | 10 |
|  | Cycling |  |  | X |  |  |  | X |  |  | X |  |  |  |  |  |  | 3 |
|  | Swimming |  |  |  |  |  |  | X |  |  | X |  |  |  |  |  |  | 2 |
|  | Resistance Training (body weight, resistance bands) |  |  |  |  |  |  |  |  |  |  |  |  |  |  |  | X | 1 |
|  | Weight Training (must include weights) |  |  |  |  |  |  |  |  |  |  |  |  |  |  |  |  | 0 |
| Benefits of exercise during period | |  |  |  |  |  |  |  |  |  |  |  |  |  |  |  |  |  |
|  | Improve physical symptoms (e.g., back pain, cramps, or fatigue) | X | X | X | X | X | X | X | X | X | X | X | X | X | X | X | X | 16 |
|  | Improves mental symptoms (mood swing, stress, or depression) | X | X | X | X |  | X | X | X | X | X | X | X | X | X | X | X | 15 |
|  | Benefits located within the exercise instructions, demonstrations, workout videos. |  |  |  |  |  |  |  |  |  |  |  |  |  |  |  |  |  |
|  | Yes |  | X | X | X | X | X | X | X | X | X | X | X | X |  |  | X | 12 |
|  | No | X |  |  |  |  |  |  |  |  |  |  |  |  | X | X |  | 3 |
| Contraindications to exercise | |  |  |  |  |  |  |  |  |  |  |  |  |  |  |  |  |  |
|  | Yes | X | X | X | X | X | X | X | X | X | X | X | X | X | X | X | X | 16 |
|  | No |  |  |  |  |  |  |  |  |  |  |  |  |  |  |  |  | 0 |
| Recommendations on exercise (e.g., stay hydrated, avoid inadequate nutrition, avoid physical activity / exercise at high altitude) during period. | |  |  |  |  |  |  |  |  |  |  |  |  |  |  |  |  |  |
|  | Yes | X |  | X |  |  | X |  | X |  |  |  | X | X |  |  |  | 6 |
|  | No |  | X |  | X | X |  | X |  | X | X | X |  |  | X | X | X | 10 |
