# Supplementary material for: Mobile applications available in Saudi Arabia for the management of Primary Dysmenorrhea: A quality review and content analysis
Source: PLoS One. 2025 Jun 12;20(6):e0325652. doi: 10.1371/journal.pone.0325652 (PMC12161552; doi:10.1371/journal.pone.0325652)
Supplement: Attachment — Submitted filename: Response_to_Reviewer_Comments_auresp_2.docx [file pone.0325652.s006.docx]

**Response to Reviewer’s Comments**

**Manuscript Title**: Mobile applications available in Saudi Arabia for the management of Primary Dysmenorrhea: a quality review and content analysis
**Manuscript ID**: PONE-D-24-49548R1
**Journal**: PLOS ONE

Dear Editor,

We sincerely thank you and the reviewer for the valuable feedback provided on our manuscript. We have carefully considered all comments and substantially revised the manuscript to address each point raised. Below is a detailed, point-by-point response to Reviewer #1’s comments. All additions and modifications have been incorporated into the revised manuscript and are highlighted using track changes.

|  | **Reviewer’s Comments** | **Our Responses** | **Page #** |
| --- | --- | --- | --- |
| 1 | You provide complete answers to my questions, yet make hardly any significant changes in the manuscript. The explanations you give me should be given to the reader and, therefore, be part of the paper | We thank the reviewer for this important reminder. We have now integrated all previously provided explanations directly into the manuscript in relevant sections (Introduction, Rationale, Methods, and Discussion). These additions include references and clearer argumentation regarding the study’s rationale, scope, and contribution. Every major reviewer point is now explicitly and transparently addressed in the manuscript body. | - |
| 2 | I still don't understand your question. Are you looking at the quality of apps or the effectiveness of exercise? | We have reworded both the abstract and the final paragraph of the introduction to clarify the dual aim of the study. Specifically, we now state that our study evaluates: (1) the overall quality of MTAs using the Mobile Application Rating Scale (MARS), and (2) the alignment of exercise-related content with established physical therapy guidelines, particularly the FITT (Frequency, Intensity, Time, and Type) principle. We do not claim to assess the clinical efficacy of the apps or their exercise interventions. | Page 7 |
| 3 | Why focus on PD? The answer you give is descriptive, not analytical. | We have added a new paragraph in the “Dysmenorrhea and its Management” section to justify our focus on primary dysmenorrhea (PD). Our rationale includes its high prevalence (up to 89%), its suitability for non-invasive self-management, and the methodological advantage of studying a condition that does not require medical diagnosis. These points are now referenced and contextualized within the research literature. | Page 4 |
| 4 | Provide references and evidence for your explanations… You say exercise helps with PD but give no evidence. | We have added multiple references to support the use of exercise-based interventions for primary dysmenorrhea. These citations are now included in the “Physical Therapy Approach for PD” section, which highlights the evidence-based rationale for evaluating exercise content in mHealth apps for PD management. | Page 4 |
| 5 | Why focus on exercise? Please give solid arguments supported by literature. | A new paragraph has been added at the end of the “Physical Therapy Approach for PD Management” section. This explains that exercise-based strategies are evidence-backed, safe, and cost-effective, and are already included in many non-pharmacological treatment protocols for PD. Supporting references have been added. | Page 4 |
| 6 | PD isn’t mentioned later. How did you ensure these apps were only for PD and not secondary dysmenorrhea? | We have now included a methodological clarification in the “Eligibility Criteria” section explaining that we excluded apps referring to endometriosis, fibroids, or other pathological pelvic conditions. Our inclusion criteria relied on keyword searches and app descriptions that specified menstrual pain or cramps without underlying medical conditions. | Page 9 |
| 7 | Why Arabic? You did not exclude anything based on the Arabic nature of your study. | We have clarified this point in the Methods and Limitations sections. While the intent was to include Arabic-language apps, our search strategy did not use Arabic-only language as an exclusion criterion. The majority of high-rated apps in Saudi Arabia are in English, and we included them to reflect actual user availability and experience. We now clearly state that this is a limitation in serving monolingual Arabic speakers and highlight it as a gap in the market. | Page 10, page 23 |
| 8 | What is your conclusion? I don’t understand what I should have learned. | We have rewritten both the opening paragraph of the Discussion section and the Conclusion. These now clearly summarize the dual findings: that most MTAs offer acceptable app quality but fail to meet clinical standards for exercise content. We emphasize the need for interdisciplinary development, localization, and evidence-based integration in MTA design for managing PD. The implications for app developers, physical therapists, and women’s health stakeholders are now clearly articulated. | Page 23 |

We hope that the revisions made in response to your insightful feedback have strengthened our manuscript and clarified our contributions to this important area of mHealth research. We are grateful for the reviewer’s careful and constructive critique, which has significantly enhanced the clarity, scientific rigor, and relevance of our work.

We respectfully submit the revised manuscript for your further consideration.

Sincerely,
Dr. Tahani J. Alahmadi
On behalf of all authors
Princess Nourah bint Abdulrahman University
Email: [Tjalahmadi@pnu.edu.sa](mailto:Tjalahmadi@pnu.edu.sa)
