# Supplementary material for: Mobile applications available in Saudi Arabia for the management of Primary Dysmenorrhea: A quality review and content analysis
Source: PLoS One. 2025 Jun 12;20(6):e0325652. doi: 10.1371/journal.pone.0325652 (PMC12161552; doi:10.1371/journal.pone.0325652)
Supplement: Attachment — Submitted filename: Response to Reviewer comments.docx [file pone.0325652.s005.docx]

**Response to Reviewer comments**

**Authors would like to thank the reviewers for their genuine and sincere comments on our study. We have considered all the comments respectfully and tried our best to address them in the best possible manner. Changes in the manuscript are carried out in review mode to highlight the additions or deletions.**

**Reviewer #1**: You wrote about a relevant paper addressing a possible opportunity to increase women's Health in Saudi Arabia. It was a well-written paper which was pleasant to read.

My main issue is that I am missing a concrete research question and hypothesis. Usually, the introduction ends with a research question, but yours does not. What is your research question, and why is this relevant?
However, I found a few additional issues difficult to distil from the paper, which might increase the paper's quality and better address your point.

**1. Why are you focusing on physical exercises after period tracking? You wrote quite a long introduction, but I don't find any solid arguments as to why exercising should be part of the period tracker. Many lifestyle interventions influence dysmenorrhea (e.g. diets, sleep, etc). Why do you want to narrow your evaluation to only exercises?**

**Response 1:** Dear Sir, Thank you so much for your valuable insights. Physical therapy approaches, particularly exercise-based interventions, have demonstrated significant effectiveness in reducing primary dysmenorrhea pain. Both low-intensity exercises like yoga and high-intensity exercises such as aerobics have shown positive results in alleviating menstrual pain intensity. Exercise is highlighted as a safe, affordable, and cost-effective method for managing PD symptoms, making it a practical alternative to pharmaceutical treatments that often come with side effects. Specific exercise regimens, including 45 to 60 minutes of exercise three times a week with activities like yoga, aerobic exercises, stretching, Kegel exercises, and relaxation techniques, have been found to effectively improve PD symptoms. While other lifestyle interventions are acknowledged, exercise-based interventions are emphasized as particularly advantageous due to their safety, affordability, and effectiveness in managing PD symptoms. This is we focused on the evaluation of exercise programs in the apps.

**2. Why are to focusing on primary dysmenorrhea? There are no good arguments why period tracking, combined with exercises, is only effective for primary dysmenorrhea and not, for example, for endometriosis. Please elaborate on why you limit to primary dysmenorrhea.**

**Response 2:** Our research limits the evaluation to primary dysmenorrhea because it is defined by the absence of pelvic abnormalities, predominantly affects young women, and allows for a more focused and consistent methodological approach. Although other lifestyle interventions may impact various conditions, the effectiveness of period tracking combined with exercise is evaluated specifically in the context of primary dysmenorrhea due to its high prevalence and the clarity of its diagnostic criteria.

**3. Why limit to Arabic? I understand that you want to evaluate the effect on Arabic culture, but most of your apps are in English only. I don’t understand how your methodology addressed the Arabic problems. It would make more sense if you only used Arabic apps, but you didn’t. So why does your study not apply to all English-speaking countries? You did not exclude anything based on the Arabic nature of your study, did you?**

**Response 3:** We appreciate the valid critique because there appears to be a disconnect between the stated focus on Arabic culture/users and the actual methodology and app selection. This is one of the limitations of our research. The study doesn't present clear evidence of why its findings would be specific to Arabic contexts when the apps evaluated were predominantly in English. The lack of Arabic language support in the apps suggests the findings might be more applicable to English-speaking populations. This suggests that the study's results could be more broadly applicable to English-speaking population of the country. Dear Reviewers, as you know the study was carried out by a group of students, and since no study is perfect, we have addressed your comments by modifying our limitation section.

4. you introduce the relevance of primary dysmenorrhea, but you don't mention it anymore in the paper in the results or discussion sections. How did you evaluate the apps for primary dysmenorrhea only? How did you know if the app was used for secondary dysmenorrhea either? Please explain the role of primary dysmenorrhea in the evaluation of the apps in the results of method section.
**Response 4:** We thank you for this valuable comment. Our research focuses basically on the evaluation of the Mobile apps and it was not aimed at distinguishing between primary and secondary dysmenorrhea. As mentioned in the previous comment, our study was explicitly designed for evaluation of MTAs aimed at managing primary dysmenorrhea. This was established by selecting only those apps that, based on their descriptions, were intended to help manage menstrual symptoms—specifically those relevant to PD. The focus on PD comes from its definition: painful menstrual cramps in women with no identifiable pelvic pathology, a condition that predominantly affects young women. Moreover, management of secondary dysmenorrhea requires diagnosis followed by physicians’ consultation. Hence, it is understood by the description of the apps and the understanding of the diseases that the selected app users are affected by PD. In addition, since our approach was only to evaluate those MTAs which are designed to manage PD, we didn’t mention further discussion of PD elsewhere by default.

5. Finally, what is your conclusion? This links back to your question. What is the answer to your question, based on the results? I don’t understand what I should have learned from your paper and how that can influence future research. It seems now that your conclusion is mainly for app developers, but your readers are mainly researchers, not developers.

**Response 5:** The conclusion has been revised and clarified. In a nutshell, this study answers its research question by emphasizing that while MTAs are a promising tool for menstrual health management, current versions fall short in delivering evidence-based exercise content for PD. This gap opens several avenues for future research aimed at enhancing clinical effectiveness, user engagement, and cultural relevance, thereby ultimately improving women's health outcomes. We are sure that our data will help the researchers in coming across potential challenges, and these information with these researchers carve the pathways for developers of MTAs.

**Reviewer #2:** Overall, the manuscript presents a well-executed study with significant contributions to the field of mHealth and women’s health. The methodology is rigorous, and the findings are well supported by data. However, certain areas require further refinement. The research question should be more explicitly stated in the introduction, and justification for methodological choices, such as the exclusion of lower-rated apps, should be provided. Greater emphasis on inter-rater reliability would enhance the validity of the app evaluations. The discussion would benefit from more direct comparisons with prior studies, and the results could be presented more effectively with visual aids. The conclusion should incorporate broader policy implications and clearly define future research directions.

**Response: Dear Sir, thank you so much for taking time to review and provide feedback that is insightful. Your comments definitely helped us improve our manuscript. We made following changes:**

1. The research question has been stated at the end of the introduction.
2. The exclusion criteria have been explained in methods.
3. Since our study is first of its kind in comparing the MTAs for PD, we are short of comparative studies to be included in the discussion section.
4. Graphical representation of mean app quality score has been done.
